# Evidence for More than One Parkinson's Disease-Associated Variant within the *HLA* Region

**DOI:** 10.1371/journal.pone.0027109

**Published:** 2011-11-09

**Authors:** Erin M. Hill-Burns, Stewart A. Factor, Cyrus P. Zabetian, Glenys Thomson, Haydeh Payami

**Affiliations:** 1 New York State Department of Health Wadsworth Center, Albany, New York, United States of America; 2 Department of Neurology, Emory University School of Medicine, Atlanta, Georgia, United States of America; 3 Veteran's Affairs Puget Sound Health Care System and Department of Neurology, University of Washington, Seattle, Washington, United States of America; 4 Department of Integrative Biology, University of California, Berkeley, California, United States of America; Oslo University Hospital, Norway

## Abstract

Parkinson's disease (PD) was recently found to be associated with *HLA* in a genome-wide association study (GWAS). Follow-up GWAS's replicated the PD-*HLA* association but their top hits differ. Do the different hits tag the same locus or is there more than one PD-associated variant within *HLA*? We show that the top GWAS hits are not correlated with each other (0.00≤r^2^≤0.15). Using our GWAS (2000 cases, 1986 controls) we conducted step-wise conditional analysis on 107 SNPs with P<10^−3^ for PD-association; 103 dropped-out, four remained significant. Each SNP, when conditioned on the other three, yielded P_SNP1_ = 5×10^−4^, P_SNP2_ = 5×10^−4^, P_SNP3_ = 4×10^−3^ and P_SNP4_ = 0.025. The four SNPs were not correlated (0.01≤r^2^≤0.20). Haplotype analysis (excluding rare SNP2) revealed increasing PD risk with increasing risk alleles from OR = 1.27, P = 5×10^−3^ for one risk allele to OR = 1.65, P = 4×10^−8^ for three. Using additional 843 cases and 856 controls we replicated the independent effects of SNP1 (P_conditioned-on-SNP4_ = 0.04) and SNP4 (P_conditioned-on-SNP1_ = 0.04); SNP2 and SNP3 could not be replicated. In pooled GWAS and replication, SNP1 had OR_conditioned-on-SNP4_ = 1.23, P_conditioned-on-SNP4_ = 6×10^−7^; SNP4 had OR_conditioned-on-SNP1_ = 1.18, P_conditioned-on-SNP1_ = 3×10^−3^; and the haplotype with both risk alleles had OR = 1.48, P = 2×10^−12^. Genotypic OR increased with the number of risk alleles an individual possessed up to OR = 1.94, P = 2×10^−11^ for individuals who were homozygous for the risk allele at both SNP1 and SNP4. SNP1 is a variant in *HLA-DRA* and is associated with *HLA-DRA, DRB5 and DQA2* gene expression. SNP4 is correlated (r^2^ = 0.95) with variants that are associated with *HLA-DQA2* expression, and with the top *HLA* SNP from the IPDGC GWAS (r^2^ = 0.60). Our findings suggest more than one PD-*HLA* association; either different alleles of the same gene, or separate loci.

## Introduction

The recent discovery of an association between PD and *HLA* was made in a hypothesis neutral genome-wide association study (GWAS) [1]. Historically, *HLA*-disease associations have been conducted with the highly polymorphic “classical” *HLA* loci; i.e., those that encode the diversity for antigen recognition; whereas, the association peak in our GWAS was in *HLA-DRA* which is practically monomorphic and hence not normally investigated for disease associations. The finding that genetic variants in immune response affect risk of developing PD, firmly grounds, at the DNA level, the long held notion that the immune system and inflammation play a significant role in PD [2].

Our original GWAS that uncovered an association between PD and *HLA* was performed with the NeuroGenetic Research Consortium (NGRC) data. NGRC is a single data set (2000 persons with PD, 1986 control volunteers) which was collected using uniform protocols for all study procedures including subject selection and diagnosis, data collection, genotyping and data analysis [1]. The NGRC GWAS revealed a spike at *HLA* for association with PD that reached genome-wide significance. The association peak was at rs3129882, a SNP in intron 1 of *HLA-DRA* which had previously been shown to associate with variation in expression of *HLA-DRA, DRB5 and DQA2* [3], [4]. Association of rs3129882 with PD had an odds ratio (OR) = 1.31, and P = 3×10^−8^ in discovery and was replicated in independent datasets in the same study [1]. The *HLA* region spike in the NGRC data included 107 SNPs that achieved P values of 10^−3^ to 3×10^−8^. A subsequent GWAS conducted in the Dutch population (772 cases, 2024 controls) confirmed the association of PD with *HLA* [5]. Their most significant SNP was rs4248166: OR = 1.36, P = 4×10^−5^ which also maps to the *HLA* class II region. The involvement of the *HLA* region in PD was also confirmed by the International Parkinson Disease Genomics Consortium (IPDGC) meta-analysis [6], which identified chr6:32588205 in the *HLA* class II region as the most significant SNP in their discovery sample (5333 cases, 12019 controls) with OR = 0.70, P = 3×10^−8^ and in their replication sample (7053 cases, 9007 controls) OR = 0.80, P = 9×10^−8^.

It is not unexpected that different GWAS's are identifying different *HLA* SNPs; arrays with different SNPs were used. However, do the different top SNPs from various studies all tag the same locus or could there be more than one PD-associated susceptibility variant in *HLA*? The aim of this study was to explore this question.

## Results

### *HLA* hits in three GWAS's

**Figure 1:**
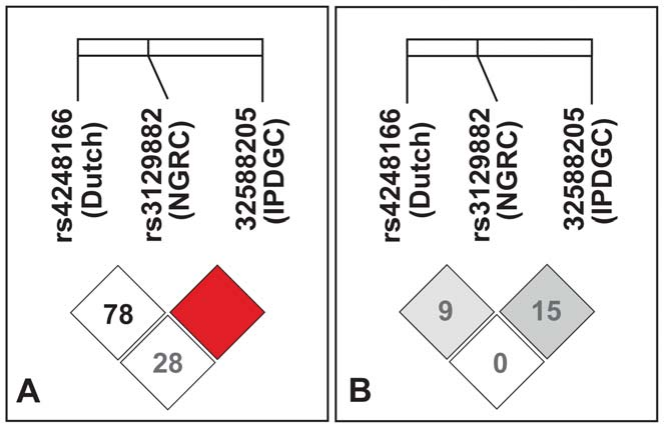
Linkage disequilibrium (LD) among top *HLA* associations from three PD GWAS's. LD was measured as D′ (panel A) and as r^2^ (panel B) among rs3129882 from the NGRC GWAS [1], rs4248166 from Dutch GWAS [5], and 6–32588205 from IPDGC meta analysis [6]. Numbers in cells are % D′ (panel A) and % r^2^ (panel B). The red cells with no number reflects D′ = 1.0.

The most significant *HLA* SNPs from the three GWAS's including and subsequent to our original report are shown in **Table 1**. They span a ∼100 kb region in the *HLA* class II region. We expected to find strong linkage disequilibrium (LD) (measured by r^2^, where r is the correlation coefficient) among them, assuming all three are tagging the same PD-susceptibility locus. Surprisingly, there was very little correlation between the SNPs as evidenced by pair wise r^2^ = 0.00, 0.09 and 0.15 (**Figure 1**).

**Table 1:** *HLA* SNPs that have shown the most significant associations with PD in three GWAS's.

GWAS	Ncases	Ncontrols	SNP	Gene	BP	MAFcases	MAFcontrols	OR	P
NGRC [1]	2000	1986	rs3129882	*DRA*	32517508	0.46	0.40	1.31	3×10^−8^
Dutch [5]	772	2024	rs4248166	*BTNL2*	32474399	0.21	0.17	1.36	4×10^−5^
IPDGC [6]	5333	12019	chr6:32588205	3′ of *DRB5*	32588205	(0.15 unspecified)	0.70	3×10^−8^	

### Conditional analysis in NGRC GWAS

To gain a better understanding of the *HLA* association with PD, we performed step-wise conditional analysis in the NGRC GWAS. Conditional analysis allows testing all the SNPs in the region (or a chosen subset, here according to statistical significance), to identify the most significant one, and repeating the analysis conditioned on the most significant SNP to see if others are significant in addition to the top SNP [7], [8], [9]. The process is repeated, each time conditioning on all SNPs that emerged as most significant in prior rounds, until all SNPs whose significance is dependent on other SNPs are identified and removed. We performed conditional analysis on 107 *HLA* SNPs that had achieved P<0.001 for PD-association in the NGRC GWAS [1], using PLINK v1.07 software [10] (**Table S1**). In round 1, when analysis was performed conditioned on the single most significant SNP (SNP1, rs3129882), 90 of 106 SNPs lost significance while 16 SNPs remained significant at P<0.05. The SNP with the lowest P value (rs3993757, P = 0.002) was marked SNP2. When the analysis was repeated (round two) conditioned on both SNP1 and SNP2 for the 15 SNPs that survived round one, 13 were associated with PD with P<0.05, and the most significant SNP of this analysis (rs2844505, P = 0.006) was marked SNP3. When the analysis was repeated with the 12 remaining SNPs conditioning now on SNP1 and SNP2 and SNP3 (round three), only one SNP had P<0.05 (rs9268515, P = 0.025) and it was marked SNP4. Summary of the results are shown in **Table 2**. For the full analysis see **Table S1**.

**Table 2:** Step-wise conditional analysis.

	SNP	BP	Minor/MajorAllele	MAFcases	MAFcontrols	HWEP	Unconditioned (GWAS results for SNPs that survived conditional analysis, see Table S1 for full data)		Conditioned on SNP1		Conditioned on SNP1 & SNP2		Conditioned on SNP1 & SNP2 & SNP3	
							OR	P	OR	P	OR	P	OR	P
SNP1	rs3129882	32517508	**G**/A	0.46	0.40	0.82	1.31	3×10^−8^						
SNP2	rs3993757	31698725	**T**/C	0.03	0.02	1.00	1.79	7×10^−4^	1.70	2×10^−3^				
SNP3	rs2844505	31547042	**G**/A	0.29	0.25	0.01	1.23	9×10^−5^	1.15	0.011	1.16	6×10^−3^		
SNP4	rs9268515	32487273	C/**G**	0.16	0.20	0.33	1.25	4×10^−4^	1.14	0.049	1.16	0.028	1.16	0.025
Number of SNPs remaining significant at the end of each round							107 with P<10^−3^ in GWAS (**Table S1**)		16 with P<0.05		13 with P<0.05		1 with P<0.05	

Step-wise conditional analysis was performed for 107 SNPs in the *HLA* region that achieved P<10^−3^ in GWAS. The full analysis is shown in **Table S1**. Here, we show the summary results for the four SNPs that remained significant after conditioning on the other significant SNPs. For consistency, we show all odds ratios (OR) on the positive side (i.e., testing risk allele against the alternate allele). The risk allele at each SNP is shown in bold. All association tests were adjusted for age at enrollment, sex, and PC1 and PC2 (principal components that define Jewish/non-Jewish origin and the European country of ancestry). Once the four SNPs that retain conditioned P<0.05 were identified, we re-tested association of each of the SNPs with PD conditioning on the other three. We obtained P = 5×10^−4^ for SNP1 conditioned on SNP2 and SNP3 and SNP4, P = 5×10^−4^ for SNP2 conditioned on SNP1 and SNP3 and SNP4, P = 4×10^−3^ for SNP3 conditioned on SNP1 and SNP2 and SNP4, and P = 0.025 for SNP4 conditioned on SNP1 and SNP2 and SNP3. BP = base pair position of the SNP on chromosome 6. Minor/major allele = the two alternative nucleotides at the SNP, the one with higher frequency denoted as major allele. MAF = minor allele frequency. HWE P = P value for the test of Hardy-Weinberg Equilibrium.

Step-wise conditional analysis revealed four *HLA* SNPs with seemingly independent effects on PD. We re-tested association of each of the four SNPs with PD conditioning on the other three. We obtained P = 5×10^−4^ for SNP1 conditioned on SNP2 and SNP3 and SNP4, P = 5×10^−4^ for SNP2 conditioned on SNP1 and SNP3 and SNP4, P = 4×10^−3^ for SNP3 conditioned on SNP1 and SNP2 and SNP4, and P = 0.025 for SNP4 conditioned on SNP1 and SNP2 and SNP3.

### Interaction among SNPs 1–4 in NGRC

We tested for and did not find significant evidence for interaction among the four SNPs. The full model testing all pair-wise interactions among the four SNPs compared with a model with no interactions yielded P = 0.9. Testing each pair-wise interaction, with all other SNPs in the model as covariates, yielded P = 0.2–0.7. Lack of evidence for interaction could have been due to insufficient power (discussed further under Replication).

### Linkage Disequilibrium

**Figure 2:**
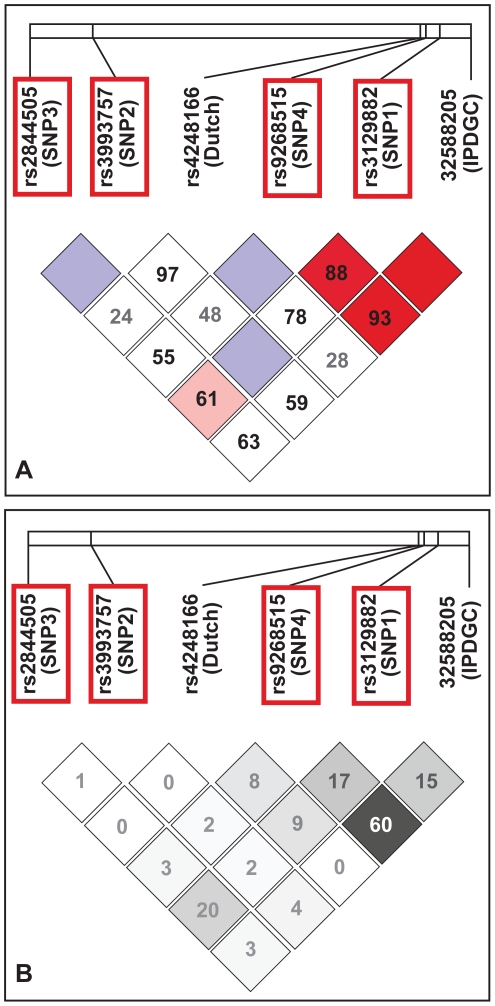
LD among the four significant SNPs from NGRC conditional analysis and the most significant HLA SNPs from other PD GWAS's. LD was measured as D′ (panel A) and as r^2^ (panel B). NGRC SNPs are numbered 1–4 and marked in red frame. The most significant *HLA* SNP from other GWAS's are indicated by rs number and the study name. Numbers in cells are % D′ (panel A) and % r^2^ (panel B). Red cell without a number reflects D′ = 1, blue cells without a number reflect D′ = 0.

We examined LD between the four SNPs that withstood conditional analysis. LD was measured as D′ and r^2^ (correlation coefficient) [11]. In the context of disease association, r^2^ is commonly used to assess correlation among SNPs (see for example [8]). Using the NGRC data for estimating LD, pair-wise D′ for the four NGRC SNPs ranged from 0.17 to 0.75 and r^2^ ranged from 0.00 to 0.09. Using the 1000 Genomes Project data for estimating LD (to allow inclusion of IPDGC SNP) the NGRC SNPs 1–4 had D′ = 0 to 0.88 and r^2^ = 0.01 to 0.20 (**Figure 2**). In relation to the top SNPs from other GWAS's (**Figure 2**), SNP1, SNP2, and SNP3 showed little or no correlation with them (0.00≤r^2^≤0.15), whereas SNP4 was moderately correlated with the top SNP from IPDGC (r^2^ = 0.60).

### Haplotype analysis

We performed haplotype analysis for the SNPs that had emerged from NGRC conditional analysis (SNP1, SNP3 and SNP4; SNP2 was not included because its minor allele was rare and none of the haplotypes carrying the minor allele of SNP2 had a frequency above 0.01) (**Table 3**). We set the haplotype that carries the lower risk allele for each SNP (AAC) as the reference and calculated relative effects of each haplotype on PD risk. (High risk alleles are shown in bold for ease of reading). The high risk allele was the minor allele for SNP1 and SNP3, and the major allele for SNP4. The haplotype with the most significant association with PD was **GGG** which has the high risk alleles at all three SNPs (OR = 1.65, 95%CI = 1.38–1.97, P = 4×10^−8^). The next most significant haplotype was **G**A**G** with two high risk alleles at SNP1 and SNP4 (OR = 1.57, 95%CI = 1.32–1.86, P = 2×10^−7^), followed by A**GG** with two high risk alleles at SNP3 and SNP4 (OR = 1.56, 95%CI = 1.22–1.99, P = 4×10^−4^), and then AA**G** with one high risk allele at SNP4 (OR = 1.27, 95%CI = 1.07–1.50, P = 5×10^−3^). **G**AC had the highest OR estimate despite having the risk allele only at SNP1 (OR = 1.75, 95%CI = 1.07–2.87, P = 0.03); note however that the confidence interval was large due to the low frequency of this haplotype.

**Table 3:** Haplotype analysis.

Haplotypes SNP1 (rs3129882) SNP3 (rs2844505) SNP4 (rs9268515)	Freq.Cases	Freq.Controls	OR (95% CI)	P
AAC	0.11	0.15	Reference	
AA**G**	0.33	0.36	1.27 (1.07–1.50)	5×10^−3^
A**G**C	0.03	0.03	1.34 (0.93–1.91)	0.11
**G**AC	0.02	0.01	1.75 (1.07–2.87)	0.03
A**GG**	0.07	0.06	1.56 (1.22–1.99)	4×10^−4^
**GG**C	0.006	0.007	-	-
**G**A**G**	0.26	0.23	1.57 (1.32–1.86)	2×10^−7^
**GGG**	0.18	0.15	1.65 (1.38–1.97)	4×10^−8^

Haplotypes were composed of SNP1, SNP3 and SNP4, shown in that order. These SNPs and SNP2 survived conditional analysis at P<0.05. SNP2 had low frequency and any haplotype with minor allele of SNP2 was too infrequent (<0.01) to be included in haplotype association tests. For each SNP, the allele that was associated with higher risk for PD (see **Table 2**) is shown in bold. OR and P values were calculated for each haplotype relative to AAC haplotype which has the lowest risk for PD. Analyses were adjusted for age at enrollment, sex, PC1, and PC2 (principal components that define Jewish/non-Jewish origin and the European country of ancestry). AA**G** consists of major alleles for each SNP; **GG**C consists of minor alleles for each SNP. Haplotype **GG**C was not tested because its frequency was <0.01.

### Replication

We attempted to replicate the following observations: (a) SNPs 1–4 are associated with PD; (b) association of each SNP with PD remains significant when conditioned on the other three SNPs; (c) haplotype analyses will reveal similar pattern of increasing risk with increasing risk alleles as seen in our NGRC results. We performed the replication in an independent dataset (843 cases and 856 controls) that is published [12] and publicly available on the NIH database of Genotypes and Phenotypes (dbGaP, accession number phs000126.v1.p1).

We replicated the association of PD with SNP1 (rs3129882, OR = 1.20, P = 0.006) and with SNP4 (rs9268515, OR = 1.25, P = 0.004); SNP3 did not replicate (**Table 4**). SNP2 was not genotyped in that dataset and could not be imputed. SNP1 and SNP4 remained significant in replication when conditioned on each other (SNP1 OR = 1.15, P = 0.04; SNP4 OR = 1.19, P = 0.04, **Table 4**).

**Table 4:** Replication of conditional analysis.

			Minor/MajorAllele*	Replication 843 cases, 856 controls									Pooled NGRC & Replication 2843 cases, 2842 controls								
				MAFCase	MAFControl	HWEP	Unconditioned		Conditioned on SNP1		Conditioned on SNP4		MAFCase	MAFControl	HWEP	Unconditioned		Conditioned on SNP1		Conditioned on SNP4	
	SNP	BP					OR	P	OR	P	OR	P				OR	P	OR	P	OR	P
SNP1	rs3129882	32517508	**G**/A	0.45	0.41	0.69	1.20	0.006	-	-	1.15	0.04	0.46	0.40	0.98	1.28	3×10^−10^	-	-	1.23	6×10^−7^
SNP3	rs2844505	31547042	**G**/A	0.26	0.24	0.18	1.04	0.32	-	-	-	-	-	-	-	-	-	-	-	-	-
SNP4	rs9268515	32487273	C/**G**	0.20	0.23	0.29	1.25	0.004	1.19	0.04	-	-	0.17	0.21	0.96	1.28	2×10^−6^	1.18	3×10^−3^	-	-

*Odds ratios (OR) were calculated for the risk allele (shown in bold).

In the replication dataset, SNP1 and SNP3 were genotyped, SNP2 was not genotyped and did not impute, SNP4 was imputed (info score = 0.95). We used imputed genotype probabilities in the R software, adjusting for age and sex for replication, and adjusting for age, sex and study for pooled data. Replication P values are one sided [28] given the directionality of the hypothesis; P values for pooled NGRC and replication are two sided.

We performed haplotype analysis for SNP1 and SNP4. Since the NGRC haplotype analysis was performed with three SNPs, for a direct comparison, we repeated the NGRC haplotype analysis with SNP1 and SNP4, leaving out SNP3. The NGRC and replication haplotype analyses yielded similar results (**Table 5**), with the most significant effect for the haplotype **GG** with two risk alleles (OR_NGRC_ = 1.51, P_NGRC_ = 1×10^−9^, OR_Replication_ = 1.41, P_Replication_ = 2×10^−4^, OR_Pooled_ = 1.48, P_Pooled_ = 2×10^−12^), followed by A**G** (OR_NGRC_ = 1.23, P_NGRC_ = 3×10^−3^, OR_Replication_ = 1.28, P_Replication_ = 0.01, OR_Pooled_ = 1.25, P_Pooled_ = 2×10^−4^); the results for **G**C are less reliable due to the smaller sample size (**Table 5**).

**Table 5:** Haplotype association test of SNP1 (rs3129882) and SNP4 (rs9268515) with PD in NGRC and Replication.

Haplotype SNP1 (rs3129882) SNP4 (rs9268515)	NGRC				Replication				Pooled NGRC + Replication			
	Freq incasesN = 4000	Freq incontrolsN = 3972	OR(95%CI)	P	Freq incasesN = 1686	Freq incontrolsN = 1712	OR(95%CI)	P	Freq incasesN = 5686	Freq incontrolsN = 5684	OR(95% CI)	P
AC	0.14	0.18	reference		0.17	0.22	Reference		0.15	0.19	reference	
**G**C	0.02	0.02	1.59(1.01–2.51)	0.05	0.02	0.02	1.71(0.85–3.44)	0.07	0.02	0.02	1.63(1.11–2.39)	0.01
A**G**	0.40	0.43	1.23(1.07–1.42)	3×10^−3^	0.38	0.38	1.28(1.04–1.57)	0.01	0.40	0.41	1.25(1.11–1.40)	2×10^−4^
**GG**	0.44	0.38	1.51(1.32–1.72)	10^−9^	0.43	0.39	1.41(1.16–1.71)	2×10^−4^	0.44	0.38	1.48(1.33–1.65)	2×10^−12^

OR and P values were calculated for each haplotype relative to the low-risk haplotype (AC) for association with PD. N = number of chromosomes. The bolded alleles are associated with higher risk. Freq = haplotype frequency. P values are two sided for NGRC, one-sided for replication.

Test of interaction between SNP1 and SNP4 in the combined NGRC and replication data yielded OR_Interaction_ = 1.12, P_Interaction_ = 0.19. If interaction exists, it is weak (OR_interaction_∼1.12) and would require a larger sample size than available here to achieve significance. Our power to detect OR_interaction_ = 1.12 with P = 0.05 was 42%. We had 80% power to detect OR_interaction_≥1.20, and 90% power to detect OR_interaction_≥1.23, using the combined NGRC and replication data. Therefore, we should have been able to detect interactions with moderate or high magnitude.

The effects of SNP1 and SNP4 appeared to be additive, as suggested by the OR for genotypic combinations, increasing incrementally with the number of risk alleles an individual possessed up to OR = 1.94, P = 2×10^−11^ for individuals who were homozygous for the risk allele at both SNP1 and SNP4 (**Table 6**).

**Table 6:** Additive effects of SNP1 and SNP4 genotypes.*

SNP1 genotype & SNP4 genotype	N case	N control	OR (95% CI)	P
AA & C_§	362	535	Ref	ref
AA & **GG**	439	469	1.37 (1.24–1.51)	0.001
A**G** & C_	436	461	1.42 (1.28–1.56)	4×10^−4^
A**G** & **GG**	959	865	1.61 (1.48–1.76)	2×10^−8^
**GG** & C_	47	38	1.78 (1.41–2.26)	0.01
**GG** & **GG**	531	414	1.94 (1.76–2.14)	2×10^−11^

*NGRC and replication are pooled.

§ The C allele of SNP4 is rare; therefore we combined CC and CG genotypes and denoted as C_.

N = number of individuals with each genotype combination. Risk alleles are shown in bold. Tests were adjusted for age and sex.

## Discussion

It is intriguing that different GWAS's have identified different SNPs as their top PD associated SNP in the *HLA* region. We questioned whether they tag the same susceptibility locus. In this study, we demonstrated that the top hits from different studies are *not* strongly correlated with each other. Low correlation among PD-associated SNPs does not rule out the possibility that they tag the same locus, but it does raise the possibility that there may be more than one PD signal in *HLA*. Using step-wise conditional analyses on the NGRC data we uncovered four seemingly independent signals for PD within the *HLA* region. There was no correlation and no detectable interaction among the four NGRC SNPs. Two of the four NGRC SNPs, rs3129882 (SNP1) and rs9268515 (SNP4), were replicated in an independent dataset. We have therefore demonstrated that there are at least two *HLA*-PD associations that cannot be explained by LD. These signals may indicate different PD-associated alleles at a single susceptibility gene, or different PD susceptibility loci. In support of the different alleles in the same gene hypothesis, there are known examples of multiple disease-associated alleles at the same gene for *HLA*-associated diseases including type1 diabetes [13] and multiple sclerosis [14], as well as multiple association signals for PD within one gene, notably, *SNCA* [8], [15]. Alternatively, the signals may represent different genes. SNP1, rs3129882, the original genome-wide significant finding in NGRC, is an expression quantitative trait locus (eQTL). It is in intron 1 of *HLA-DRA* and is associated with expression levels of *HLA-DRA*, *DRB5* and *DQA2* genes [3], [4]. SNP4, rs9268515, is highly correlated (r^2^ = 0.95) with three eQTL SNPs (rs3793127, rs3763309, rs3763312) that are associated with expression levels of *HLA-DQA2* [4]. SNP4 also correlates with the top SNP of IPDGC (r^2^ = 0.60), which maps between *DRA* and *DRB5*, and could be tagging any of the classical *HLA* genes in the *DR*-*DQ* region due to their high LD. This raises the intriguing possibility that PD may be associated with both regulatory elements that influence HLA class II gene expression, and with a classical *HLA* class II allele.

The allele frequency for SNP1 varies significantly in Caucasian Americans according to the European country from which their ancestors immigrated to the US [1]. Furthermore, SNP1 is more strongly associated with sporadic PD than familial PD [1]. Therefore, depending on subject selection, a study may find a positive association (as reported in NGRC), no association, or even an inverse association between SNP1 and PD. We used principal components to correct for ethnic and geographic variability. The subpopulations that differed were the Jewish and the Irish (defined by self-report and verified by principal components [1]). SNP1 and SNP4 remained significant when we excluded the Jewish and the Irish individuals. A recent study showed an inverse association between SNP1 and PD in a mixed Irish and Polish population [16]. It is noteworthy that in the original NGRC GWAS report [1], the Americans of Irish-descent also showed an inverse association between SNP1 and PD, while all other European-descent subpopulations showed a positive association (number of individuals of Polish-descent were few). The Irish/Polish study found the association using a recessive model, as compared to additive model used in NGRC. This is also in agreement with the NGRC data, because the recessive model projects a larger effect size than additive model because it compares individuals who are homozygous for the risk allele to all others.

SNP2 and SNP3 are more than 800 kb away from SNP1 and SNP4. SNP2 had a low minor allele frequency and thus could not be studied in any detail. SNP3 indicated a potential third signal in the NGRC data, but it did not replicate. Much larger sample sizes will be required to determine if and how SNP2 and SNP3 affect susceptibility to PD.

SNP4 was the last of the four seemingly independent SNPs to show up in the conditional analysis of the NGRC data with a modest P = 0.025; and it was the only one of the four NGRC SNPs that showed any correlation with the top *HLA* hit of IPDGC. The direction of the SNP4 effect was the same as the IPDGC SNP; they reported reduced risk with the minor allele, we report increased risk with the major allele (we used the risk alleles throughout the text and the main tables to keep the ORs consistently in the positive direction). We used a liberal P<0.05 given the exploratory nature of the study and that we would follow with a replication study. That our last significant *HLA* SNP tagged the most significant *HLA* SNP in IPDGC gives credence to SNP4 being a true association rather than a false positive signal due to a relaxed significance threshold. SNP4 was genotyped in NGRC and imputed in the replication study. The Impute Information score was 0.95, suggesting relatively high reliability. Cases and controls were imputed together under the same conditions, using the same method, and independently of NGRC, and the results showed significantly different allele frequencies between cases and controls for SNP4, in line with the original observation in NGRC. Thus it is unlikely that the results for replication were severely skewed by the fact that SNP4 was imputed; however, replication by other studies will help clarify further.

We do not know if *HLA* plays a larger role for PD risk for certain individuals and perhaps little or no role for others. *HLA* may be involved in a subtype of PD; for example, some cases of PD may be due to infection [17] or autoimmunity [18]. Alternatively, *HLA* may have a ubiquitous role in all PD perhaps via inflammatory response to a variety of causes [19]. The two possibilities are not mutually exclusive; i.e., it is possible that *HLA* and the immune system affect PD pathogenesis in more than one way.

Our results illustrate the utility of conditional analyses and examination of LD structure in replication studies. These exploratory studies can be used to investigate the possibility of more than one disease association in a region. They can also help understand the differences in the results across association studies. We acknowledge the exploratory nature of this study and the difficulty of deciphering *HLA*-disease association due to the complex structure of the region. At this point, we have probably identified markers and not the true risk alleles. Not only is it critical to understand the nature of the association(s) of PD with *HLA*, it is equally important to understand the interrelationship between PD and other *HLA*-associated disorders, particularly multiple sclerosis which like PD is a neurodegenerative disorder [14], [20], [21], [22]. Solving this evolving story will take open collaboration to amass large datasets with genotype, sequence, expression and epigenetic data.

## Materials and Methods

### Human Subjects

This study was approved by the Institutional Review Boards of the New York State Department of Health, Emory University and Atlanta VA Medical Center, VA Puget Sound Heath Care System and University of Washington, and Albany Medical College. All participants gave consent. The majority was signed written consent. A subset of participants who preferred to remain anonymous gave verbal consent. Both written and verbal consent procedures were approved by the IRBs and documented in each participant's data file. A GWAS conducted with 2000 cases and 1986 controls from the NGRC, which had previously identified *HLA* as a PD-associated gene [1] was examined as the primary dataset here. Persons with PD had been diagnosed using standard criteria [23]. Cases and controls were unrelated, non-Hispanic Caucasians from the United States. (See below for Replication dataset.)

### Genotyping

DNA was obtained from whole blood for NGRC subjects, and from blood, cell lines, or whole genome amplified DNA for the replication dataset. NGRC individuals were genotyped using the Illumina HumanOmni1-Quad_v1-0_B array, with a call rate of 99.92% and 99.99% reproducibility. Details of the GWAS genotyping and statistical quality control (QC) have been previously published [1]. 9,232 SNPs were genotyped in the *HLA* region in NGRC (from 29–33 Mb on chromosome 6; genome build 36).

### Quality Control for NGRC GWAS

NGRC genome-wide genotypes had previously been filtered based on standard QC criteria [1]. Two principal components (PC1 and PC2) were found to associate significantly with PD, representing Jewish/non-Jewish ancestry and European countries of ancestral origin. Sex also significantly associated with case/control status because PD is more prevalent in men than in women, and the population used in this study reflects that disparity. Furthermore, while controls in NGRC were intentionally selected to be older than patients' age at onset, age effects are not completely corrected by the study design. Thus, all association tests with NGRC data included age at enrollment, sex, PC1, and PC2 as covariates.

### Step-wise conditional Analysis

The step-wise conditioning followed a previously described and commonly used protocol [7], with the modification that here we used logistic regression instead of chi-square and adjusted for covariates (sex, age, PC1, PC2). We used 107 *HLA* SNPS that reached P<0.001 in GWAS. For round 1 of the conditional analysis, all 107 SNPs were ranked by P value. The SNP with lowest P value was marked as SNP1. The remaining 106 SNPs were tested for association with PD risk conditioned on SNP1. The SNP with the lowest P value was marked SNP2, and analysis was repeated now conditioning on SNP1 and SNP2. The process was repeated until no remaining SNPs had P<0.05 for association with PD. We chose to use P<0.05, rather than a more stringent threshold, because the study was exploratory and would be followed with replication.

### Linkage Disequilibrium

We used Haploview [24] to construct haplotypes, visualize haploblocks, and estimate LD and r^2^. Since not all of the SNPs reported by other studies were genotyped or imputed in NGRC, LD was calculated using genotypes from the 1000 Genomes Project [25]. SNP genotypes were downloaded from the 1000 Genomes Project website (www.1000genomes.org; CEU low coverage, July 2010 release).

### Haplotype Analysis

We used HAPSTAT-3.0 [26] to construct the haplotypes, estimate haplotype frequencies for the top associating SNPs and test haplotype association with PD, while adjusting for age, sex, PC1 and PC2. Only haplotypes with frequencies of ≥0.01 in cases and controls combined were included.

### Replication

The dataset for replication was downloaded from dbGaP with IRB approval and Data Use Certification from the National Institute of Neurological Disorders and Stroke (NINDS) ((http://www.ncbi.nlm.nih.gov/sites/entrez?db=gap) accession number phs000126.v1.p1). The replication dataset was a single GWAS with 843 persons with PD collected by the PROGENI and GenePD studies and 856 neuro-normal controls from the NINDS Repository [12]. Cases were familial PD (one individual per family). Diagnosis was made using standard criteria. Cases and controls were Caucasian and unrelated. SNP1 (rs3129882) and SNP3 (rs2844505) were genotyped. We imputed SNP4 (rs9268515) with information score = 0.95. SNP2 was not genotyped and did not impute. IMPUTE v2 [27] was used with HapMap 3 and 1000 Genomes Project genotypes as reference data. Genotype probabilities (dose 2-0) were analyzed in R software http://www.r-project.org/. Haplotype analyses were performed as described above. P values were one-sided for replication given the directional hypotheses [28]. The pooled analyses of NGRC and replication had two-sided P values and were adjusted for study as well as age and sex.

### Interaction

The full model for testing all pair-wise interactions among the four SNPs in NGRC was [SNP1+SNP2+SNP3+SNP4+SNP1*SNP2+SNP1*SNP3+SNP1*SNP4+SNP2*SNP3+SNP2*SNP4+SNP3*SNP4+covariates] vs. [SNP1+SNP2+SNP3+SNP4+covariates]. We also tested each pair of SNPs one at a time, while keeping all other SNPs in the model as covariates. For example, for interaction between SNP1 and SNP2 the model was [SNP1*SNP2+SNP1+SNP2+SNP3+SNP4+covariates]. All analyses included the following covariates: sex, age, PC1 and PC2. In the pooled dataset (NGRC plus replication), we tested for interaction between SNP1 and SNP4 only as [SNP1*SNP4+SNP1+SNP4+covariates]; here the covariates were age and sex. Power calculation for interaction was performed using Quanto v1.2.4 [29] with 2843 cases and 2842 controls and a two-sided α = 0.05 to estimate power of our study to detect the observed interaction OR, and to estimate the minimum interaction OR detectable with 80% or 90% power.

### Data access

NGRC data are available at www.ncbi.nlm.nih.gov/gap study accession number phs000196.v2.p1. The replication dataset was obtained from the NINDS Database found at (http://www.ncbi.nlm.nih.gov/sites/entrez?db=gap) through dbGaP accession number phs000126.v1.p1.

## Supporting Information

Table S1 **Step-wise conditional analysis including 107** ***HLA*** **SNPs that achieved P<10^−3^ in GWAS.**
(PDF)
