# Supplementary material for: Evidence for More than One Parkinson's Disease-Associated Variant within the HLA Region
Source: PLoS One. 2011 Nov 9;6(11):e27109. doi: 10.1371/journal.pone.0027109 (PMC3212531; doi:10.1371/journal.pone.0027109)
Supplement: Table S1 — Step-wise conditional analysis including 107 HLA SNPs that achieved P<10−3 in GWAS. (PDF) [file pone.0027109.s001.pdf]

Table S1. Step-wise conditional analysis including 107 *HLA* SNPs that achieved P<10^{-3} in GWAS.

In this Table, odds ratios (OR) were calculated by comparing the minor allele to the major allele. (In the Tables of the main text, the ORs were calculated comparing the risk allele to the alternate allele in order to keep all OR in the text consistently in the positive direction. Tests were adjusted for age at enrollment, sex, and PC1 and PC2 (principal components that define Jewish/non-Jewish origin and the European country of ancestry).

	SNP	BP	Minor/ Major Alleles	MAF cases	MAF controls	HWE P	Unconditioned (GWAS results)		Conditioned on SNP1		Conditioned on SNP1 & SNP2		Conditioned on SNP1 & SNP2 & SNP3	
							OR	P	OR	P	OR	P	OR	P
**SNP1**	rs3129882	32517508	G/A	0.46	0.40	0.82	1.31	**3x10^{-8}**						
**SNP2**	rs3993757	31698725	T/C	0.03	0.02	1.00	1.79	7x10^{-4}	1.70	**0.002**				
**SNP3**	rs2844505	31547042	G/A	0.29	0.25	0.01	1.23	9x10^{-5}	1.15	**0.011**	1.16	**0.006**		
**SNP4**	rs9268515	32487273	C/G	0.16	0.20	0.33	0.80	4x10^{-4}	0.88	**0.049**	0.86	**0.028**	0.86	**0.025**
	rs3117098	32466491	C/T	0.33	0.28	0.74	1.27	3x10^{-6}	1.06	0.455	1.05	0.540	1.01	0.889
	rs3129955	32473818	T/C	0.33	0.28	0.79	1.27	4x10^{-6}	1.05	0.501	1.04	0.591	1.01	0.951
	rs3117102	32458085	G/A	0.33	0.28	0.77	1.27	4x10^{-6}	1.05	0.509	1.04	0.600	1.00	0.962
	rs3129954	32473558	A/G	0.33	0.28	0.68	1.27	4x10^{-6}	1.05	0.509	1.04	0.599	1.00	0.964
	rs3117104	32456287	A/T	0.33	0.28	0.77	1.27	4x10^{-6}	1.05	0.505	1.04	0.596	1.00	0.957
	rs3129948	32462622	C/A	0.33	0.28	0.74	1.27	4x10^{-6}	1.05	0.536	1.04	0.629	1.00	0.990
	rs6923504	32536164	G/C	0.33	0.29	0.42	1.24	3x10^{-5}	1.03	0.643	1.01	0.855	0.99	0.843
	rs6903608	32536263	C/T	0.33	0.29	0.44	1.24	3x10^{-5}	1.03	0.647	1.01	0.859	0.99	0.838
	rs9268880	32539336	T/G	0.34	0.29	0.32	1.23	4x10^{-5}	1.03	0.684	1.01	0.896	0.98	0.804
	rs9268882	32539601	T/C	0.34	0.29	0.32	1.23	4x10^{-5}	1.03	0.684	1.01	0.896	0.98	0.804
	rs9269110	32551247	A/C	0.33	0.29	0.32	1.23	5x10^{-5}	1.03	0.714	1.01	0.928	0.98	0.768
	rs2050188	32447875	G/A	0.37	0.32	0.81	1.22	5x10^{-5}	0.99	0.904	0.99	0.845	0.96	0.554
	rs9275578	32787362	T/G	0.20	0.24	0.78	0.80	1x10^{-4}	0.90	0.111	0.90	0.092	0.89	0.058
	rs9275593	32788821	A/G	0.20	0.24	0.78	0.80	1x10^{-4}	0.90	0.111	0.90	0.092	0.89	0.058
	rs28435656	31988616	A/G	0.19	0.16	1.00	1.28	1x10^{-4}	1.15	**0.043**	1.17	0.023	1.09	0.283
	rs9275582	32788048	T/C	0.20	0.24	0.78	0.80	1x10^{-4}	0.90	0.111	0.90	0.092	0.89	0.058
	rs9275579	32787437	A/T	0.21	0.24	0.85	0.80	1x10^{-4}	0.90	0.106	0.90	0.090	0.89	0.061
	rs3957145	32786325	A/G	0.21	0.24	0.89	0.81	2x10^{-4}	0.91	0.114	0.90	0.097	0.89	0.063
	rs9275595	32789333	C/T	0.20	0.24	0.75	0.80	2x10^{-4}	0.91	0.124	0.90	0.103	0.89	0.066
	rs3129768	32703061	G/T	0.21	0.17	0.33	1.26	2x10^{-4}	1.08	0.310	1.10	0.200	1.04	0.598
	rs2395163	32495787	C/T	0.19	0.23	0.05	0.80	2x10^{-4}	0.90	0.094	0.89	0.065	0.88	0.051
	rs9271640	32700178	T/C	0.21	0.18	0.33	1.26	2x10^{-4}	1.07	0.326	1.09	0.214	1.04	0.631
	rs9275523	32782972	A/C	0.21	0.24	0.86	0.81	2x10^{-4}	0.91	0.118	0.90	0.100	0.89	0.066
	rs9275580	32787440	G/A	0.21	0.24	0.89	0.81	2x10^{-4}	0.91	0.119	0.90	0.101	0.89	0.066
	rs7197	32520558	T/C	0.19	0.16	0.79	1.27	2x10^{-4}	1.07	0.381	1.09	0.261	1.04	0.648
	rs9268543	32492779	T/A	0.14	0.18	0.23	0.78	2x10^{-4}	0.87	0.052	0.86	**0.030**	0.86	**0.026**
	rs2844508	31544478	C/G	0.34	0.31	0.74	1.21	2x10^{-4}	1.14	**0.012**	1.16	**0.006**	1.07	0.519
	rs9275522	32782948	T/C	0.21	0.24	0.89	0.81	2x10^{-4}	0.91	0.129	0.90	0.110	0.89	0.072
	rs9268627	32513799	C/T	0.19	0.23	0.02	0.80	2x10^{-4}	0.90	0.121	0.89	0.086	0.89	0.066
	rs9275555	32785066	T/C	0.21	0.24	0.52	0.81	2x10^{-4}	0.91	0.139	0.91	0.120	0.89	0.079
	rs3130283	32246523	A/C	0.16	0.13	0.45	1.28	3x10^{-4}	1.12	0.125	1.14	0.075	1.05	0.533
	rs9269081	32549078	A/C	0.32	0.28	0.55	1.21	3x10^{-4}	1.00	0.951	0.98	0.744	0.95	0.479
	rs17577980	32467799	A/G	0.11	0.08	0.13	1.34	3x10^{-4}	1.16	0.091	1.16	0.096	1.17	0.071
	rs7452864	32547055	T/C	0.32	0.28	0.55	1.21	3x10^{-4}	0.99	0.922	0.98	0.717	0.95	0.460
	rs3130279	32220604	A/G	0.16	0.13	0.38	1.27	3x10^{-4}	1.12	0.120	1.14	0.076	1.05	0.560
	rs3130287	32158522	C/T	0.16	0.13	0.32	1.27	3x10^{-4}	1.12	0.107	1.14	0.067	1.05	0.539
	rs2507964	31566136	A/G	0.31	0.34	0.13	0.83	3x10^{-4}	0.88	**0.015**	0.89	**0.026**	0.93	0.221
	rs2395175	32513004	A/G	0.15	0.19	0.21	0.79	3x10^{-4}	0.88	0.072	0.87	0.044	0.86	0.036
	rs9264532	31342360	C/T	0.34	0.30	0.02	1.20	3x10^{-4}	1.12	**0.030**	1.13	**0.024**	1.06	0.349
	rs3129888	32519704	G/A	0.21	0.19	0.35	1.24	4x10^{-4}	1.04	0.644	1.06	0.467	1.01	0.927
	rs3998158	32789970	C/T	0.19	0.23	0.50	0.81	4x10^{-4}	0.91	0.159	0.91	0.133	0.89	0.085
	rs7382085	32522398	G/A	0.24	0.20	0.71	1.23	4x10^{-4}	1.02	0.817	1.04	0.596	0.99	0.915
	rs3134954	32179871	G/A	0.16	0.13	0.29	1.27	4x10^{-4}	1.12	0.122	1.14	0.078	1.05	0.584
	rs2516415	31567721	T/C	0.31	0.34	0.11	0.83	4x10^{-4}	0.88	**0.015**	0.89	**0.028**	0.93	0.225
	rs9268628	32513843	C/A	0.18	0.15	0.002	1.25	4x10^{-4}	1.06	0.432	1.03	0.651	1.07	0.388
	rs2395153	32453573	G/C	0.37	0.41	0.37	0.84	4x10^{-4}	0.97	0.566	0.96	0.511	0.96	0.494
	rs9268435	32454469	A/G	0.37	0.41	0.39	0.84	4x10^{-4}	0.97	0.568	0.96	0.512	0.96	0.495
	rs2239802	32519824	G/C	0.23	0.20	0.52	1.23	4x10^{-4}	1.01	0.858	1.04	0.634	0.99	0.873
	rs2076536	32447326	G/A	0.39	0.34	3x10^{-4}	1.19	5x10^{-4}	1.02	0.805	1.01	0.919	0.99	0.809
	rs2395182	32521295	G/T	0.23	0.20	0.49	1.23	5x10^{-4}	1.01	0.850	1.04	0.626	0.99	0.881
	rs3134943	32255739	A/G	0.15	0.13	0.22	1.27	5x10^{-4}	1.11	0.159	1.13	0.103	1.05	0.583
	rs3134603	32233980	T/C	0.15	0.13	0.84	1.27	5x10^{-4}	1.12	0.144	1.14	0.090	1.05	0.551
	rs7196	32520549	A/T	0.23	0.20	0.46	1.23	5x10^{-4}	1.01	0.862	1.04	0.637	0.99	0.873
	rs3131283	32227876	A/G	0.15	0.13	0.38	1.27	5x10^{-4}	1.11	0.167	1.13	0.110	1.04	0.629
	rs9268522	32489421	T/A	0.20	0.24	0.07	0.82	5x10^{-4}	0.91	0.122	0.90	0.088	0.89	0.073
	rs3130342	32188124	T/G	0.16	0.13	0.28	1.26	5x10^{-4}	1.11	0.148	1.13	0.096	1.04	0.671
	rs2516417	31567615	C/G	0.31	0.34	0.07	0.84	5x10^{-4}	0.88	**0.018**	0.89	**0.032**	0.94	0.255
	rs3817973	32469089	A/G	0.40	0.44	0.60	0.85	5x10^{-4}	0.98	0.736	0.98	0.698	0.97	0.584
	rs9268839	32536750	G/A	0.41	0.45	0.27	0.85	6x10^{-4}	0.96	0.473	0.96	0.513	0.97	0.557
	rs2076530	32471794	G/A	0.41	0.45	0.60	0.85	6x10^{-4}	0.99	0.828	0.98	0.776	0.97	0.655

rs9270984	32681969	T/G	0.18	0.15	0.77	1.25	6x10^{-4}	1.06	0.446	1.08	0.332	1.01	0.914
rs3132940	32269374	A/C	0.15	0.13	0.50	1.27	6x10^{-4}	1.10	0.218	1.12	0.142	1.03	0.691
rs9271055	32683347	G/A	0.18	0.15	0.60	1.25	6x10^{-4}	1.06	0.454	1.08	0.338	1.01	0.923
rs3129750	32689493	G/T	0.18	0.15	0.60	1.25	6x10^{-4}	1.06	0.454	1.08	0.338	1.01	0.923
rs9378212	32553669	T/C	0.41	0.45	0.38	0.85	6x10^{-4}	0.96	0.500	0.97	0.540	0.97	0.587
rs9270986	32682038	A/C	0.18	0.15	0.73	1.25	6x10^{-4}	1.06	0.453	1.07	0.337	1.01	0.922
rs13211921	32550814	G/T	0.41	0.45	0.44	0.85	6x10^{-4}	0.96	0.483	0.96	0.522	0.97	0.567
rs1964995	32557389	G/A	0.41	0.45	0.38	0.85	7x10^{-4}	0.96	0.490	0.97	0.529	0.97	0.575
rs2844507	31544560	A/G	0.26	0.23	0.01	1.20	7x10^{-4}	1.13	**0.035**	1.14	**0.021**	0.88	0.414
rs2395037	31568279	G/A	0.38	0.42	0.43	0.85	7x10^{-4}	0.90	**0.028**	0.90	**0.047**	0.96	0.435
rs3129852	32505762	G/A	0.22	0.19	0.84	1.22	7x10^{-4}	1.02	0.826	1.04	0.625	1.00	0.968
rs9268633	32514451	A/G	0.22	0.19	0.84	1.22	7x10^{-4}	1.02	0.826	1.04	0.625	1.00	0.968
rs9394099	32557138	T/G	0.41	0.45	0.40	0.85	7x10^{-4}	0.96	0.498	0.97	0.537	0.97	0.585
rs3135334	32510664	G/A	0.22	0.19	0.84	1.22	7x10^{-4}	1.02	0.824	1.04	0.631	1.00	0.966
rs3132946	32298006	A/G	0.15	0.13	0.32	1.26	7x10^{-4}	1.09	0.239	1.11	0.158	1.03	0.713
rs4373382	32458846	C/A	0.40	0.45	0.56	0.85	7x10^{-4}	0.99	0.808	0.98	0.770	0.97	0.654
rs4424066	32462406	G/A	0.40	0.44	0.58	0.85	7x10^{-4}	0.99	0.811	0.98	0.772	0.97	0.655
rs1044506	32280043	A/C	0.15	0.13	0.55	1.26	7x10^{-4}	1.09	0.236	1.11	0.155	1.03	0.713
rs9268455	32457750	T/C	0.20	0.23	0.06	0.82	7x10^{-4}	0.91	0.117	0.90	0.085	0.90	0.083
rs9268451	32457064	C/T	0.20	0.23	0.07	0.82	8x10^{-4}	0.91	0.116	0.90	0.085	0.90	0.083
rs3793127	32479893	A/C	0.20	0.23	0.07	0.82	8x10^{-4}	0.91	0.119	0.90	0.087	0.90	0.085
rs3763309	32483951	T/C	0.20	0.23	0.07	0.82	8x10^{-4}	0.91	0.119	0.90	0.087	0.90	0.085
rs12055599	31720555	C/A	0.03	0.02	4x10^{-4}	1.73	8x10^{-4}	1.65	**0.002**	1.14	0.866	1.18	0.835
rs2076533	32471505	G/A	0.40	0.44	0.60	0.85	8x10^{-4}	0.99	0.829	0.98	0.791	0.98	0.673
rs2076529	32471933	A/G	0.40	0.44	0.60	0.85	8x10^{-4}	0.99	0.829	0.98	0.791	0.98	0.673
rs9268472	32463583	A/G	0.40	0.44	0.56	0.85	8x10^{-4}	0.99	0.820	0.98	0.781	0.97	0.663
rs3131294	32288124	T/C	0.15	0.13	0.50	1.26	8x10^{-4}	1.09	0.242	1.11	0.159	1.03	0.734
rs3134749	31327448	A/G	0.41	0.45	0.38	0.85	8x10^{-4}	0.90	**0.025**	0.89	**0.021**	0.93	0.150
rs9268460	32459261	C/T	0.40	0.44	0.56	0.85	8x10^{-4}	0.99	0.852	0.99	0.813	0.98	0.693
rs1048709	32022914	A/G	0.20	0.17	0.41	1.23	8x10^{-4}	1.11	0.091	1.13	0.054	1.06	0.409
rs9268433	32453869	G/T	0.20	0.23	0.06	0.82	8x10^{-4}	0.91	0.128	0.90	0.094	0.90	0.092
rs2904592	31568035	G/A	0.38	0.42	0.45	0.85	9x10^{-4}	0.90	**0.035**	0.91	0.057	0.96	0.507
rs3129883	32518115	T/C	0.27	0.24	0.53	1.20	9x10^{-4}	0.96	0.580	0.99	0.840	0.96	0.561
rs3763312	32484326	A/G	0.20	0.23	0.06	0.82	9x10^{-4}	0.91	0.129	0.90	0.095	0.90	0.093
rs9271775	32702306	C/T	0.19	0.16	0.96	1.23	9x10^{-4}	1.05	0.539	1.07	0.374	1.01	0.914
rs1800684	32259972	T/A	0.15	0.13	0.25	1.26	9x10^{-4}	1.10	0.228	1.11	0.154	1.02	0.780
rs28570051	31318580	T/C	0.41	0.45	0.42	0.85	9x10^{-4}	0.90	**0.027**	0.89	**0.022**	0.93	0.156
rs3129886	32518554	T/C	0.27	0.24	0.53	1.20	9x10^{-4}	0.96	0.551	0.98	0.806	0.95	0.532
rs3763317	32484766	T/C	0.43	0.46	0.59	0.85	9x10^{-4}	0.92	0.123	0.92	0.107	0.92	0.103
rs3096695	32177784	G/C	0.15	0.13	0.21	1.25	9x10^{-4}	1.10	0.195	1.12	0.132	1.03	0.745
rs3130285	32134235	T/C	0.15	0.13	0.15	1.25	9x10^{-4}	1.10	0.189	1.12	0.128	1.03	0.741
rs28490179	32626983	C/T	0.18	0.15	0.03	1.23	0.001	1.04	0.559	1.06	0.427	1.00	0.949
rs3095245	31321881	G/A	0.41	0.45	0.40	0.85	0.001	0.90	**0.030**	0.90	**0.025**	0.93	0.165
